# Structure-Guided Repurposing of Approved Drugs Identifies Aprepitant and Mavorixafor as Putative δ-Opioid Receptor Agonist Candidates

**DOI:** 10.3390/ijms27093823

**Published:** 2026-04-25

**Authors:** Rocco Buccheri, Carlo Reale, Alessandro Coco, Carmela Parenti, Lorella Pasquinucci, Antonio Rescifina

**Affiliations:** Department of Drug and Health Sciences, University of Catania, Viale A. Doria 6, 95125 Catania, Italy; rocco.buccheri@unict.it (R.B.); carlo.reale@phd.unict.it (C.R.); alessandro.coco@phd.unict.it (A.C.); carmela.parenti@unict.it (C.P.)

**Keywords:** molecular docking, virtual screening, conformational dynamics, drug repurposing, analgesia

## Abstract

δ-opioid receptor (DOR) is a promising therapeutic target for developing safer treatments for pain and neuroprotection. In this study, we applied a structure-guided drug-repurposing workflow to identify FDA-approved drugs with predicted DOR-binding and agonist-like structural features. Using a validated GNINA-based docking protocol with an active-state DOR model (PDB ID: 6PT3), we screened 2342 approved compounds and identified 39 candidates with predicted submicromolar binding affinities. These hits were further evaluated through molecular dynamics simulations, binding pocket volume analysis, and principal component analysis, which enabled the prioritization of two leading candidates, aprepitant and mavorixafor. Both compounds formed stable receptor-ligand complexes, maintained persistent interactions with Asp128, promoted contraction of the orthosteric pocket, and retained favorable redocking scores on the MD-refined receptor conformations. Overall, these results identify aprepitant and mavorixafor as promising putative DOR agonists and provide a rational foundation for their experimental validation through binding, functional, and in vivo pain studies in the future.

## 1. Introduction

δ-opioid receptor (DOR) is one of the three main opioid receptor subtypes, along with the µ-opioid receptor (MOR) and κ-opioid receptor (KOR), and belongs to the class A G-protein-coupled receptor (GPCR) superfamily. Although historically less studied than MOR, the principal target of morphine and most clinically used opioids, DOR has emerged as a highly relevant pharmacological target because of its distinctive physiological and therapeutic profiles. In contrast to MOR, DOR activation can produce analgesic effects with a substantially lower risk of respiratory depression, constipation, and dependence, making it an attractive candidate for developing safer opioid-based therapies [1,2,3]. This safer pharmacological profile has positioned DOR as an appealing target in the search for next-generation opioid analgesics. In addition to analgesia, DOR has been implicated in several additional processes of pharmacological interest. Experimental evidence supports its involvement in anxiolytic- and antidepressant-like effects [4,5], particularly in clinical settings in which chronic pain and mood disorders co-exist. DOR has also attracted attention in the context of neuroprotection and neurodegeneration, suggesting its possible therapeutic relevance in disorders such as Alzheimer’s and Parkinson’s diseases [6,7]. Further studies have linked DOR modulation to migraine and headache mechanisms, with the potential advantage of avoiding the vascular adverse effects associated with triptans [8,9]. In addition, DOR participates in the regulation of immune and inflammatory responses [10,11]. The broad therapeutic potential of DOR is consistent with its widespread distribution in both central and peripheral tissues, including regions involved in pain modulation and affective processing [12]. Functionally, DOR primarily couples to Gi/Go proteins, thereby reducing neuronal excitability and neurotransmitter release [13]. Depending on the agonist, receptor activation may also promote β-arrestin recruitment, receptor internalization, and downstream signaling through kinase pathways, such as ERK1/2 and PI3K/Akt, highlighting the complexity of DOR pharmacology and the potential relevance of biased agonism. Notably, DOR is often retained intracellularly under basal conditions and translocates to the plasma membrane in response to chronic inflammation, prolonged agonist exposure, or pathological pain states [14]. The analgesic efficacy of DOR agonists has been demonstrated, particularly in models of chronic, inflammatory, and neuropathic pain, often with limited evidence of tolerance or dependence, even after repeated administration [15,16,17]. Moreover, the simultaneous modulation of DOR and MOR may enhance analgesic efficacy while allowing for reductions in conventional opioid doses [18]. Together, these findings support the view that DOR is a promising target for opioid-sparing therapeutic strategies.

Despite this strong pharmacological rationale, the clinical translation of DOR agonists remains limited. Early compounds, such as SNC80, showed high potency but were hampered by poor pharmacokinetic properties and proconvulsant effects at high doses [19]. More recent candidates, including ADL5859, ADL5747, and KNT-127, have shown improved safety profiles; however, none have yet achieved regulatory approval [20]. These limitations underscore the need for alternative, more efficient strategies to identify clinically viable DOR modulators.

Drug repurposing is an attractive approach in this context. By identifying new therapeutic applications for approved drugs, repurposing can reduce development time, cost, and translational risk, as candidate compounds already possess known safety, pharmacokinetic, and manufacturing profiles [21]. This strategy is especially relevant in light of the polypharmacological nature of many approved drugs, which may harbor previously unrecognized activities relevant to DOR modulation [22]. Simultaneously, advances in structural biology have made high-resolution active-state models of DOR available, including structures when bound to agonists, such as those deposited under PDB IDs 6PT3 and 6PT2 [23,24]. These structures provide a robust framework for structure-based virtual screening, enabling the rapid identification of compounds with predicted DOR-binding and agonist-like interaction patterns. Molecular dynamics (MD) simulations can be used to assess the stability of ligand-receptor complexes and characterize their conformational effects on the orthosteric binding pocket [25]. The integration of virtual screening and MD simulations into drug repurposing pipelines can improve prioritization efficiency and reduce the experimental burden associated with early-stage screening [26,27]. This approach is particularly valuable for identifying approved compounds with potential translational relevance and previously unrecognized receptor-modulating properties [28,29]. Because approved drugs already have established safety and pharmacokinetic profiles, identifying FDA-approved compounds with previously unrecognized affinities for DOR may have direct translational relevance. In addition, computational repurposing may help reveal previously unappreciated pharmacodynamic activities of established medications. Therefore, the present study aimed to apply a structure-guided computational repurposing strategy to identify approved compounds with potential agonist-like activity for DOR.

## 2. Results and Discussion

### 2.1. Protein Model Validation

The protein model was validated using the workflow established in our previous studies [30]. The selected model was a DOR protein co-crystallized with the agonist ligand DPI-287 (PDB ID: 6PT3). To ensure optimal structural quality, the model was retrieved from PDB-REDO, which provides refined coordinates with improved geometry compared to those of the original PDB entry. The protein resolution was 3.30 Å, and the experimentally determined ligand inhibition constant (*K*_i_) was 0.39 nM [23]. Before molecular docking, the co-crystallized ligand was removed from the binding site, and redocking was performed using the GNINA docking algorithm. GNINA integrates convolutional neural networks (CNNs) into its scoring function to enhance the prediction accuracy. It employs a Monte Carlo (MC) sampling approach to explore the ligand conformational space through random transformations, including translations, rotations, and torsional adjustments. Local energy minimization was performed after each MC step, and the best-scoring conformations were retained for subsequent analysis. Depending on the user settings, CNNs can be applied at multiple stages of the docking pipeline. In the “rescoring” mode, GNINA uses a specified CNN model to evaluate and rank each ligand conformation according to its predicted binding quality. GNINA provides two key metrics for assessing binding affinity: CNN affinity, which estimates the expected dissociation constant (p*K*_d_) of the ligand-protein interaction, and CNN score, an internal metric reflecting the quality of the generated binding pose. The product of these two metrics, referred to as CNN_VS, is recommended by the GNINA authors as a more reliable predictor of the interaction p*K*_d_ than the individual metrics. Once the predicted p*K*_d_ was obtained, it was converted to the corresponding *K*_d_ in nanomolar units using Equation (1).

(1) $$K_{d}=10^{\left(9-pK_{d}\right)}$$

**Figure 1:**
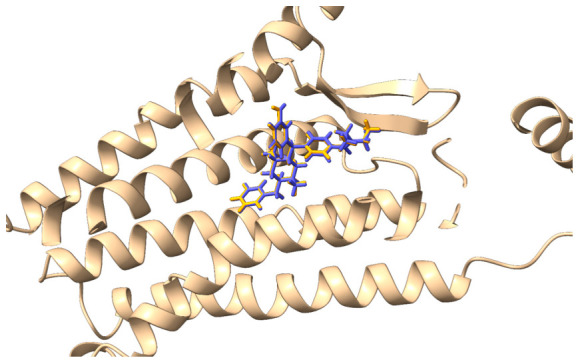
Comparison between the co-crystallized ligand pose (orange) and the reproduced pose using GNINA (blue).

The constant 9 is a scaling factor used to convert the dissociation constant from molar (M) to nanomolar (nM) units. Following redocking, the RMSD value between the experimental (co-crystallized) and predicted ligand poses was calculated. The resulting RMSD of 0.16 Å demonstrated excellent agreement, confirming that the docking algorithm successfully reproduced the experimental binding conformation (Figure 1).

Furthermore, the predicted *K*_d_ obtained from GNINA was 11.22 nM, which remained within the nanomolar range and was therefore considered consistent with a high-affinity binding estimate, although it did not quantitatively reproduce the experimental value. Together with the excellent redocking accuracy, this result supports the suitability of the docking procedure for prospective virtual screening. To further assess the predictive validity of both the docking algorithm and selected protein model, statistical validation was conducted by generating a receiver operating characteristic (ROC) curve and calculating the enrichment factors (EFs). For this analysis, a compound set of 420 molecules was assembled, comprising 20 ligands (Table 1, Figure S1) with experimentally confirmed DOR agonist activity reported in the literature and 400 decoy molecules. Decoys were designed to be structurally similar to the active compounds but lacked experimental evidence of biological activity. Nonetheless, it cannot be entirely ruled out that a small subset of decoys may retain a residual affinity for the target receptor.

**Figure 2:**
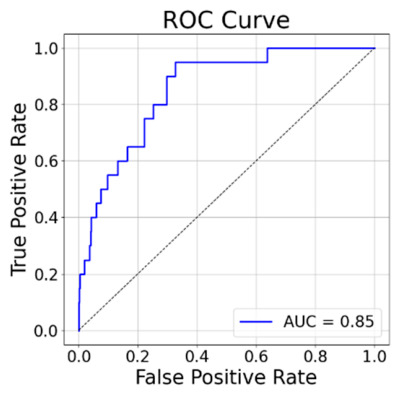
ROC curve and calculated AUC of the DOR.

VS was performed on the complete dataset, and the resulting ligand rankings were evaluated using the CNN_VS metrics. Although the CNN_VS model outputs *K*_d_ values and the experimental data are reported as *K*_i_, these quantities are not assumed to be strictly identical. Rather, they were considered sufficiently comparable for the limited purpose of relative affinity ranking in the present benchmark, in line with previous computational studies that grouped *K*_i_ and *K*_d_ as closely related affinity measurements [44]. The ROC curve yielded an area under the curve (AUC) value of 0.85 (Figure 2), while the EFs at the top 1%, 5%, and 10% of the ranked compounds were 15.75, 6.00, and 5.00, respectively.

These results indicate that the model and docking method achieved a substantially higher hit rate than random selection, corresponding to AUC = 0.50 and EF = 1.0. Collectively, the RMSD, predicted *K*_d_, ROC-AUC, and EF values supported the use of the docking protocol for prospective hit prioritization in the present study.

### 2.2. Virtual Screening

Following the validation phase, virtual screening was performed on a dataset comprising 2342 FDA-approved drugs. The docking results were evaluated using the CNN_VS metric with a cutoff value of 6.30. Because CNN_VS estimates the interaction p*K*_d_, this threshold corresponds to a *K*_d_ of 500 nM, which is a commonly accepted benchmark for compounds with good biological activity [45]. Accordingly, only compounds with CNN_VS ≥ 6.30 (i.e., *K*_d_ ≤ 500 nM) were retained for further analysis. A total of 39 molecules met this criterion and were subsequently prioritized using a rule-based interaction filter designed around literature-supported DOR recognition features. The key interactions considered were as follows: (i) salt bridge and electrostatic interactions with Asp128 [23,24,46], essential for receptor recognition; (ii) hydrogen bonding with Tyr308 [23]; and (iii) hydrophobic and aromatic contacts involving Val281, Trp284, and Leu300 [23,24,46,47] (Table 2).

Candidate selection for subsequent analysis was carried out using a hierarchical prioritization scheme. Compounds were first required to retain an ionic or electrostatic interaction with Asp128, which was considered an essential recognition feature for DOR binding. The retained molecules were grouped according to the presence of additional literature-supported contacts with Tyr308, Val281, Trp284, and Leu300, and the compounds showing the most informative interaction patterns were prioritized. The CNN_VS values were subsequently used to rank the molecules within comparable interaction classes. This rule-based procedure was adopted to reduce subjective bias and to identify a focused set of compounds for downstream MD analysis.

Based on this prioritization strategy, mavorixafor, harringtonine, mequitazine, buclizine, and aprepitant were selected for further investigation because they combined favorable predicted affinity with the most relevant interaction patterns within the orthosteric site. Mavorixafor (ID: 1322) [48], a CXCR4 antagonist, was prioritized because it exhibited the highest CNN_VS value among the selected compounds (7.39) while preserving key contacts with Asp128 and Val281. Harringtonine (ID: 2074, CNN_VS = 6.63) [49], a compound with antiviral activity, was selected because it established four relevant interactions involving Asp128, Val281, Trp284, and Leu300. Mequitazine (ID: 1068) [50], an antihistamine, was retained because of its favorable predicted affinity (CNN_VS = 6.73) and its interactions with Asp128, Val281, and Tyr308. Buclizine (ID: 1702, CNN_VS = 6.44) [51], another antihistamine, was selected based on its interaction pattern, which included Asp128, Val281, Trp284, and Leu300. Aprepitant (ID: 2030) [52], an antiemetic drug, was prioritized because of its favorable predicted affinity (CNN_VS = 6.89) and ability to engage Asp128, Val281, and Leu300.

### 2.3. Molecular Dynamics and Cavity Size Evaluation

**Figure 3:**
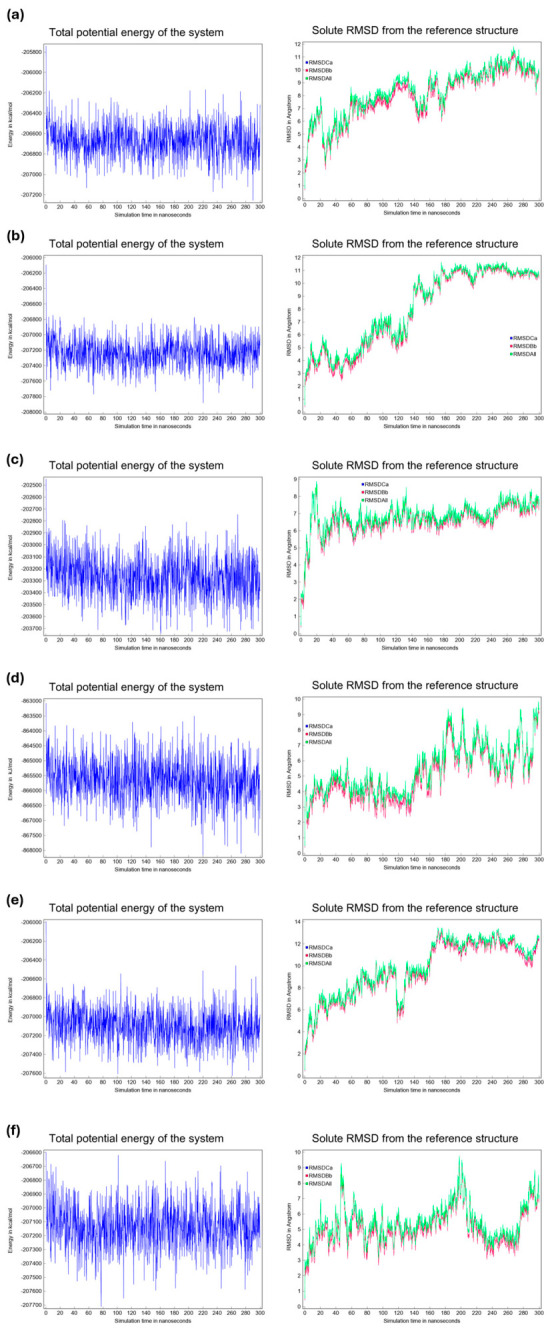
Left: Total potential energy of the system as a function of time (300 ns) for the DOR apo-like reference system and the five ligand–protein complexes. Right: Solute RMSD from the reference structure as a function of time (300 ns). Panels: (**a**) apo-like, (**b**) mequitazine, (**c**) mavorixafor, (**d**) buclizine, (**e**) harringtonine, and (**f**) aprepitant.

All-atom 300 ns MD simulations were performed for the DOR apo-like reference system and the five selected drug candidates to allow for a direct comparison of the conformational stability in a membrane environment. The simulation setup included an explicit lipid bilayer and solvent and followed the established protocols used for transmembrane GPCR simulations [53]. The potential energy and solute RMSD (from the reference structure) were monitored throughout each 300 ns trajectory to assess convergence and structural stability (Figure 3).

The potential energy traces (Figure 3, left panels) fluctuated around constant means for all systems with no systematic drift, indicating that the simulations reached a thermodynamically stable regime over the production run. The solute RMSD traces (Figure 3, right panels) displayed an initial increase during the early portion of the simulations, followed by plateau-like fluctuations for the remainder of the trajectories. The apparent equilibration interval varied slightly across the systems, with most systems reaching steady RMSD fluctuations within 20–60 ns, after which, the RMSD values fluctuated by approximately system-specific means for the remainder of the 300 ns runs. These observations collectively indicate that the protein backbone and ligand–protein complexes sampled a stable conformational ensemble throughout most of the simulations. Moreover, the analysis of representative MD snapshots confirmed that the critical electrostatic interaction with Asp128 remained stably preserved throughout the entire trajectory for all ligand-bound complexes, indicating a robust and persistent anchoring mechanism within the binding pocket.

Previous studies have shown that agonist binding to DOR is often associated with contraction of the orthosteric binding pocket [23], whereas antagonist binding is associated with pocket expansion [24]. To assess whether the ligands examined here followed this trend, we quantified the binding cavity volumes over the equilibrated portion of the trajectories using the final 20 ns of both the apo-like reference simulation and each ligand-bound MD simulation. As summarized in Table 3, all ligand-bound systems exhibited lower average pocket volumes than the apo-like reference, although to varying extents. The largest average reductions were observed for harringtonine, aprepitant, and mavorixafor, whereas buclizine and mequitazine showed moderate decreases. Because contraction of the orthosteric site has previously been associated with agonist-compatible DOR conformations, these volumetric changes were interpreted as supportive structural signatures, rather than direct evidence of receptor activation. Given the variability observed across the final trajectory window, these results should be interpreted comparatively and in conjunction with redocking and PCA analyses.

**Figure 4:**
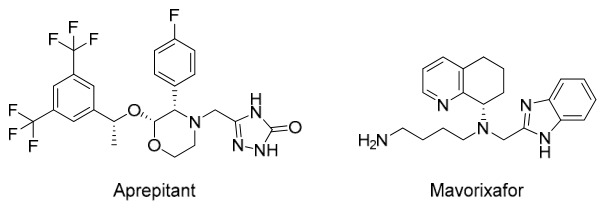
Two-dimensional structures of the two most promising putative DOR agonist candidates identified in this study.

Redocking analysis was performed on the average structure of the last 5 ns of the MD simulation. The results showed a general worsening of the calculated p*K*_d_ values for most compounds, except for aprepitant, which showed a clear and significant improvement in interaction affinity. In the case of mavorixafor, the p*K*_d_ decreased only slightly while maintaining a sufficiently promising profile for future development. Taken together, the results from all stages of this in silico investigation highlight aprepitant and mavorixafor (Figure 4) as the most promising putative DOR agonist candidates among the screened approved drug set.

The selection of aprepitant and mavorixafor is particularly noteworthy from a polypharmacological perspective. Aprepitant is an approved NK1 receptor antagonist that is currently used for the prevention of chemotherapy-induced nausea and vomiting. Given the well-established role of NK1 signaling in pain transmission and inflammation, a compound combining NK1 antagonism with putative DOR agonism could produce synergistic analgesic effects [54]. In addition, preclinical studies have suggested that NK1 receptor antagonism may attenuate opioid tolerance and reinforcement [55], thereby further complementing the intrinsically safer pharmacological profile associated with DOR-targeted therapy. Mavorixafor is a CXCR4 antagonist, and its identification is of interest because of the documented functional interplay between chemokines and opioid receptor systems. Because CXCR4 signaling is involved in neuroimmune regulation and inflammatory sensitization, a compound with combined CXCR4 antagonism and putative DOR agonism may be particularly relevant in inflammatory pain [56].

### 2.4. Principal Component Analysis of DOR Systems

**Figure 5:**
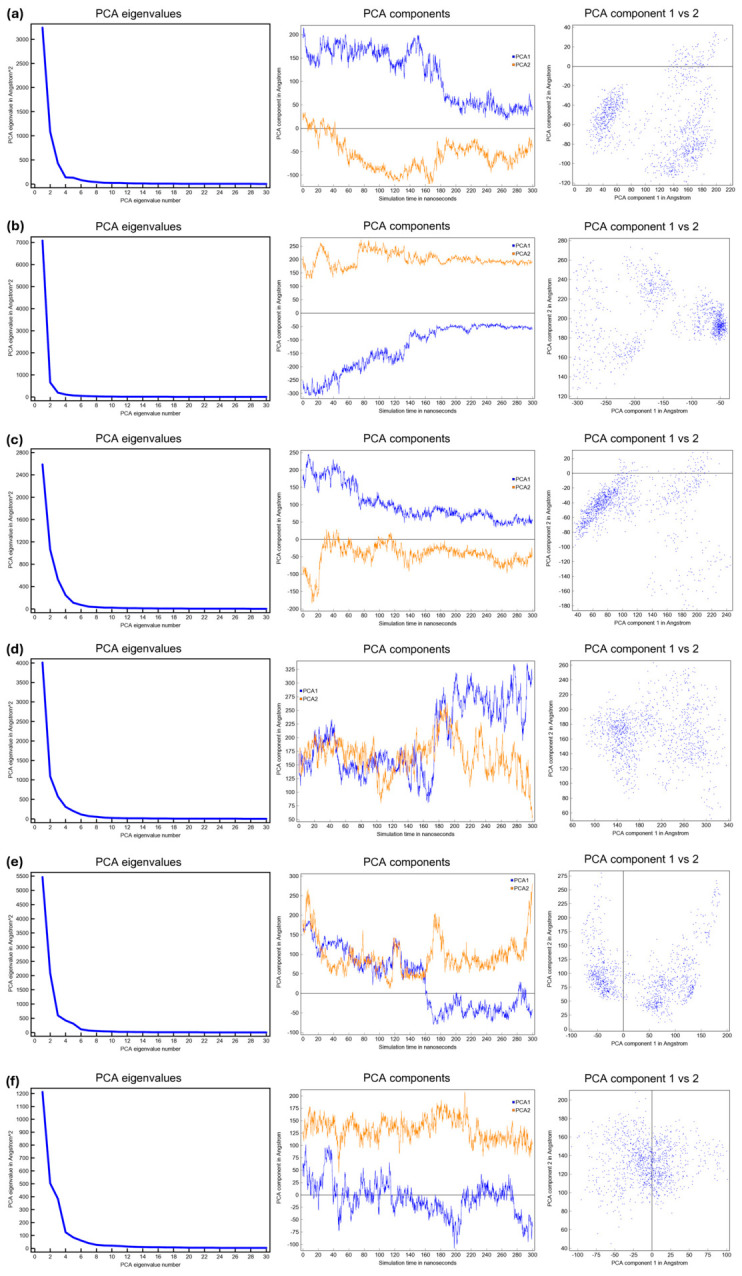
PCA of the DOR apo-like reference system (**a**), mequitazine–DOR complex (**b**), mavorixafor–DOR complex (**c**), buclizine–DOR complex (**d**), harringtonine–DOR complex (**e**), and aprepitant–DOR complex (**f**). Scree plot (left), projections of the first two principal components obtained from the 300 ns simulation (center), and projections of the 300 ns trajectory onto the first two PCA eigenvectors (right).

To characterize the collective motions and conformational dynamics of DOR in the apo state and in complex with selected ligands, principal component analysis (PCA) was performed on the 300-ns production phases of each MD trajectory. To provide a consistent apo-like reference for comparison, a ligand-removed form of the 6PT3 receptor was simulated under the same membrane, solvent, and force field conditions used for the ligand-bound systems. Figure 5 summarizes the PCA results for each system: apo-like (Figure 5a), mequitazine–DOR (Figure 5b), mavorixafor–DOR (Figure 5c), buclizine–DOR (Figure 5d), harringtonine–DOR (Figure 5e), and aprepitant–DOR (Figure 5f).

The scree plots (left column) for all systems show a steep decline in eigenvalues, indicating that the first few principal components capture the dominant, large-scale motions of the receptor. The similarity in the shape of these curves suggests that ligand binding does not dramatically reconfigure the hierarchy of collective motions, and the same leading dynamical modes are preserved across systems.

Examination of the temporal evolution of PC1 and PC2 (central column) and the corresponding 2D projections onto the first two eigenvectors (right column) revealed ligand-specific modulation of conformational sampling. The apo-like receptor (Figure 5a) sampled a broader region of the PC1–PC2 plane than most ligand-bound systems, consistent with the increased conformational heterogeneity in the absence of an orthosteric ligand. In contrast, many ligand-bound systems show attenuated fluctuations, although the magnitude of stabilization is ligand-dependent.

Mequitazine (Figure 5b) and mavorixafor (Figure 5c) display the most constrained PC trajectories and tightly clustered 2D projections, consistent with the strong confinement of the receptor conformational ensemble.

Buclizine (Figure 5d) occupies an intermediate position. Fluctuations were reduced relative to the apo state but were more pronounced than those in the mequitazine and mavorixafor systems.

Harringtonine (Figure 5e) showed the most significant fluctuations among ligand-bound complexes, particularly along PC1, and samples a comparatively broad conformational region; therefore, it exerted the weakest stabilizing effect of the ligands tested.

Aprepitant (Figure 5f) moderately attenuated fluctuations and occupied a conformational subspace between the compact mequitazine/mavorixafor clusters and the broader harringtonine/apo distributions.

In summary, the PCA results indicate that ligand binding preserves the primary hierarchy of receptor dynamic modes while selectively constraining their amplitudes and sampling in a ligand-specific manner. All ligands reduced conformational heterogeneity relative to the apo state but with markedly different magnitudes. These differences likely reflect the varying abilities of the ligands to confine the receptor within specific conformational subspaces. This may reflect the differential compatibility of conformational states associated with receptor activation.

These PCA results, when interpreted together with volumetric and redocking analyses, provide a clear basis for deprioritizing harringtonine, mequitazine, and buclizine. Although all three compounds showed a reduction in average binding pocket volume relative to the apo-like reference, their overall post-MD profiles remained less favorable than those of aprepitant and mavorixafor. In the case of harringtonine, the pronounced fluctuations along PC1 and PC2 indicate a lack of dynamic stabilization, despite the marked pocket contraction. Mequitazine, while showing a moderate reduction in pocket volume, did not display the same convergence of favorable descriptors observed for the top candidates and showed the largest decrease in the redocked CNN_VS score relative to the initial docking result. Similarly, buclizine exhibited a less pronounced volumetric effect and a clear worsening of the predicted binding affinity after MD. Taken together, these dynamic and affinity-related features support the exclusion of harringtonine, mequitazine, and buclizine and justify the prioritization of aprepitant and mavorixafor as the most robust candidates for subsequent experimental validation.

Two limitations should be considered when interpreting the results of the present study. First, the docking benchmark included a limited number of experimentally supported actives; therefore, the validation metrics are best interpreted as evidence of practical screening utility rather than exhaustive protocol generalizability. Second, the MD analyses were based on single trajectories for each system and therefore provided exploratory conformational evidence rather than replicate-supported dynamic statistics.

## 3. Materials and Methods

### 3.1. Protein Preparation

The protein was prepared using the YASARA software (v. 25.1.13, YASARA Biosciences GmbH, Vienna, Austria) by adding hydrogens using YASARA’s ‘Clean → All’ option. The free energy was minimized before docking using YASARA’s ‘Energy minimization’ option in YASARA. Finally, the co-crystallized ligand was removed using the YASARA software.

### 3.2. Dataset and 3D Structures Generation

The FDA-approved drug dataset was provided by MCE (MedChemExpress, Monmouth Junction, NJ, USA) in the SDF file format. The SDF file was cleaned in DataWarrior (v. 6.05.03, OpenMolecules, Allschwil, Svizzera) by removing salts and duplicate molecules. Ligand preparation was performed using Open Babel (v. 3.1.1) [57], where 3D structures were generated and minimized at physiological pH (7.4). The 3D structures were then optimized at the GFN2 semiempirical level using the xTB (extended tight-binding, v. 6.7.1) package [58] because published benchmarks indicate that it generally provides geometries in closer agreement with quantum-chemical reference structures than generic molecular mechanics force fields, especially for bond lengths, bond angles, and overall conformational features of organic molecules [59,60]. Geometry optimization was performed using an analytical linearized Poisson-Boltzmann (ALPB) model for water, with the charge states specified for each molecule based on the physiological pH.

### 3.3. Molecular Docking Analysis

Molecular docking analysis was performed using the molecular docking algorithm GNINA (v. 1.3) [61]. Regarding GNINA, we used the “rescore” docking mode, which involved 15 ligand pose rotations, and the poses were sorted by the CNN score. The protein input was in the PDB format, and the ligand input was in the SDF format. The simulation box was built using AutoDock Tools (v. 1.5.7, The Scripps Research Institute, La Jolla, CA, USA), with the co-crystallized ligand pose serving as a reference. The grid parameters were chosen to be sufficiently wide so as not to force the ligand-receptor interaction. The grid parameters used for docking all ligands were center x = 3.367, center y = −40.327, center z = −53.569, npts x = 24, npts y = 28, npts z = 40, and spacing = 1.

### 3.4. Decoys Generation

Decoys were generated using the LUDe web server [62] from a reference set of 20 literature-supported DOR active ligands. LUDe was applied independently to each active ligand rather than to a pooled set. For each active compound, 20 physicochemically matched decoys were generated, resulting in 400 decoys. This active-to-decoy ratio (~5% actives) was selected in accordance with previous studies showing that highly imbalanced datasets can provide a more realistic evaluation of virtual screening performance [63,64]. Decoys were selected to match the general physicochemical properties of the corresponding active ligands, while differing in two-dimensional topology. As part of the LUDe workflow, all candidate decoys were cross-checked against the complete set of 20 active ligands, and any molecule with a Tanimoto similarity > 0.2 to any active compound was excluded. The 3D structures of the retained decoys were prepared as described in Section 3.2.

### 3.5. Molecular Dynamics Simulations

MD simulations were performed using the YASARA software. The cubic simulation cell was extended by 5 Å around all protein atoms, with periodic boundary conditions in all directions, and MD was performed using the md_runmembranefast-integrated macro. The simulation was performed at physiological pH in a physiological water solution (0.9% NaCl) at 298 K, with a water density of 0.997 g/mL. For the Van der Waals forces, the cut-off was 8 Å, and no cut-off was applied to the electrostatic forces (using the Long-range Coulomb algorithm). MD simulations were performed for 300 ns using the ff14SB force field. After the MD simulation, the root mean square deviation (RMSD) and internal energy trends over time were evaluated using the md_analyze macro. The membrane composition was 90% phosphatidylcholine (POPC) and 10% cholesterol on each side, as reported previously [53]. One 300 ns production trajectory was generated for each system and used for exploratory comparative analysis. Accordingly, the MD results were interpreted as conformational prioritization data rather than statistically converged ensemble estimates.

For the apo-like reference system, the co-crystallized ligand DPI-287 was removed from 6PT3 prior to system setup, and the receptor was simulated using the same protocol as that adopted for all ligand-bound complexes.

### 3.6. Principal Component Analysis

Principal component analysis (PCA), a multivariate statistical technique, was performed on the MD trajectories of the apo-like receptor reference and the five ligand-bound systems generated from the same structural framework to enable state comparisons under matched simulation conditions using the YASARA md_analyze macro. For this purpose, a covariance matrix of the backbone atom fluctuations (N, C, and Cα) was constructed and diagonalized to extract the dominant modes of motion. PCA reduces the dimensionality of complex datasets while retaining the most relevant information, thereby facilitating the identification of large-scale conformational changes that are otherwise difficult to detect. The global dynamics of the receptor-ligand complexes were primarily assessed using the first two principal components, PC1 (projection onto eigenvector 1) and PC2 (projection onto eigenvector 2), which capture the most significant modes of collective motion and provide insights into the conformational flexibility and stability of the studied systems.

### 3.7. Binding Site Cavity Analysis

The binding site cavity size was analyzed using CAVER Analyst (v. 2.0 BETA) [65]. The ‘Cavity Computation’ option integrated into the software was used, with the settings ‘Large probe’ = 5.50 Å and ‘Probe’ = 3.10 Å. The probe settings were chosen to correctly discriminate the size and shape of the binding sites. Once the analysis was performed in the ‘Cavities Overview’ menu, the volumetric value was refined by setting ‘# Samples’ = 500,000.

### 3.8. ROC Curves and Enrichment Factors

ROC curves and related AUC values were calculated using the roc_curve() and auc() functions of the sklearn.metrics module (scikit-learn v. 1.3.0). A set of 420 compounds was prepared for each protein target, including the 20 most active ligands based on experimental affinity values and 400 decoy molecules. The active ligands represented the positive set, whereas the 400 decoys represented a presumed inactive benchmark set generated to match the physicochemical profile of the active ligands while differing in topology. The false-positive rate (FPR) and true-positive rate (TPR) were calculated by comparing the binary labels with the predicted scores of each docking method. The AUC was calculated using the trapezoidal numerical integration of the ROC curve. ROC curves were generated using Matplotlib (v. 3.7.1), plotting the TPR vs. FPR for each method, with the respective AUC values indicated in the legends. A diagonal reference line (random classifier) was included for the visual comparison of performance.

The EFs were calculated at thresholds of 1%, 5%, and 10% of the total dataset. For each predictive method, the dataset was sorted in descending order of the score. The number of active compounds in the top-ranked subset was divided by the expected number of active compounds in a random selection of the same size, according to the formula reported in Equation (2):

(2) $$EF=\frac{\left(N\_active\_top/N\_top\right)}{\left(N\_active\_totals/N\_totals\right)}$$

where N_active_top represents the number of active compounds in the selected subset, N_top represents the size of the subset, N_active_totals indicates the total number of active compounds in the dataset, and N_totals indicates the total size of the dataset.

### 3.9. RMSD Calculation

RMSD calculations were performed using the *obrms* command in Open Babel (v. 3.1.1) [57]. The co-crystallized ligand and docking output poses were provided in the PDB format.

### 3.10. Ligand-Protein Interactions Analysis

Ligand-protein interactions were analyzed using BIOVIA Discovery Studio Visualizer (v. 25.1.0.24284, Dassault Systèmes Biovia Corp., San Diego, CA, USA) software. The co-crystallized structure was uploaded in PDB format, and all hydrogen atoms were added using the ‘Chemistry → Hydrogen → Add’ option in the upper toolbar. The co-crystallized pose was selected and defined as a ligand. The interactions were then analyzed using the ‘Show 2D Diagram’ option.

### 3.11. Hit Prioritization Strategy

Following docking, compounds with CNN_VS values ≥ 6.30 were retained. Hit prioritization was then performed using a hierarchical rule-based procedure. An ionic or electrostatic interaction with Asp128 was considered mandatory for progression based on its established role in DOR ligand recognition. The retained compounds were subsequently grouped according to the presence of additional contacts with Tyr308, Val281, Trp284, and Leu300, which were considered to be informative secondary recognition features. The CNN_VS values were then used to rank the compounds within comparable interaction classes and to prioritize a reduced set of molecules for MD simulations. This procedure was adopted to minimize subjective selection bias.

## 4. Conclusions

In the present study, we applied an integrated in silico workflow based on structure-guided virtual screening and molecular dynamics simulations to explore the DOR-targeting potential of clinically approved drugs. The selected model, corresponding to the active-state δ-opioid receptor protein co-crystallized with the agonist DPI-287 (PDB ID: 6PT3), together with the GNINA-based docking protocol, showed good overall performance, as demonstrated by accurate pose reproduction in redocking experiments, satisfactory discrimination between actives and decoys, and favorable early enrichment. These results support the robustness of the proposed computational framework for prospective screening applications.

Screening of 2342 FDA-approved drugs yielded 39 compounds with predicted sub-micromolar affinity, which were subsequently prioritized according to key receptor-interaction features known to be relevant for DOR recognition. This refinement led to the selection of five candidates (mavorixafor, harringtonine, mequitazine, buclizine, and aprepitant) for further dynamic characterization. Molecular dynamics simulations indicated stable ligand–receptor complexes and persistent engagement of Asp128 across ligand-bound systems. Binding pocket volume analysis performed over the equilibrated portion of the trajectories showed that all five compounds reduced the average orthosteric-site volume relative to the apo-like reference, with the most pronounced contractions observed for harringtonine, aprepitant, and mavorixafor. Because orthosteric-site contraction has previously been associated with agonist-compatible DOR conformations, these volumetric changes were interpreted as supportive structural signatures and were considered together with the PCA and MD redocking results. Based on these findings, aprepitant and mavorixafor emerged as the most promising candidates, as they displayed the most favorable overall convergence of docking, interaction, volumetric, dynamic, and post-MD redocking descriptors.

Overall, the present findings identify aprepitant and mavorixafor as promising putative DOR agonist candidates for repurposing and provide a rational basis for their prioritization in future pharmacological studies. Importantly, the current results should be interpreted as evidence of computational prioritization rather than a definitive demonstration of functional agonism. To fully assess the repurposing hypothesis, the translational potential of these candidates must be established using a stepwise experimental validation strategy. This pipeline should begin with radioligand binding assays to confirm DOR affinity and receptor subtype selectivity, followed by functional studies, such as Gi-mediated cAMP inhibition and β-arrestin recruitment assays, to define their agonist profile more precisely. Ultimately, evaluation in neuropathic and inflammatory pain models is necessary to determine their in vivo efficacy and potential as repurposed opioid-sparing analgesics.

Taken together, this study offers a reproducible and efficient computational framework for DOR-oriented drug repurposing and highlights how approved drug libraries can be leveraged to uncover previously unrecognized opportunities in GPCR-targeted drug discovery. By narrowing the large chemical space to a small number of mechanistically plausible candidates, this study provides a focused starting point for future experimental investigations aimed at establishing whether these approved drugs can be repositioned as bona fide DOR modulators with therapeutic relevance.

## Figures and Tables

**Table 1:** Details of the 20 active ligands selected for analysis against the decoy *^a^*.

Name	*K*_i_ Experimental (nM)	Reference
Compound **21**	0.025	[31]
Oxazatricyclodecane (**2a**)	0.154	[32]
Compound **24**	0.170	[31]
Compound **23**	0.180	[31]
Compound **29**	0.221	[31]
Compound **2**	0.240	[33]
ADZ7268	0.280	[31]
DPI-287	0.390	[23]
Compound **8e**	0.420	[34]
SNC-162	0.625	[35]
TAN-67	0.647	[36]
Compound **4**	0.880	[31]
Xorphanol	1.000	[37]
AZD2327	1.200	[31]
(+)-BW373U86	1.800	[38]
ARD353	1.900	[39]
DPI-221	2.000	[40]
Compound **3h**	2.300	[41]
Compound **24**	24.000	[42]
Nalbuphine	240.000	[43]

*^a^* Compound numbering corresponds to that in the original publication. Two entries labeled “Compound 24” originated from different literature sources and retained the original numbering used in the respective publications.

**Table 2:** Intermolecular interactions identified for the 39 selected ligands during virtual screening. A value of 1 indicates the presence of the corresponding ligand–residue interaction, whereas 0 indicates its absence *^a^*.

ID	CNN_VS	Asp128	Val281	Trp284	Leu300	Tyr308
646	6.55	1	1	0	0	0
837	6.51	0	1	0	0	0
861	6.31	0	0	0	0	1
931	6.74	0	1	0	0	0
1016	6.67	0	1	0	0	0
1023	6.37	1	1	0	0	0
1041	6.36	0	0	0	0	0
1042	6.89	0	1	0	0	0
1068	6.73	1	1	0	0	1
1097	7.00	0	1	0	0	1
1102	6.56	0	1	0	0	1
1120	6.60	1	1	0	0	0
1225	6.83	0	1	0	0	1
1238	6.35	1	1	0	0	0
1283	6.60	1	1	0	0	0
1290	7.10	0	1	0	0	1
1313	6.71	0	1	0	0	0
1322	7.39	1	1	0	0	0
1351	6.48	0	1	0	1	0
1371	7.30	0	1	0	0	1
1517	6.41	1	1	0	1	0
1560	6.36	1	1	0	0	0
1618	6.86	0	1	0	0	1
1652	6.42	0	1	0	0	0
1702	6.44	1	1	1	1	0
1737	6.37	0	1	1	1	0
1738	6.34	0	1	1	1	0
1752	6.79	0	1	1	1	0
1833	6.96	0	1	1	1	1
1835	7.29	0	1	1	1	0
1923	7.07	0	1	1	1	0
1951	6.39	0	1	1	1	1
2030	6.89	1	1	0	1	0
2069	6.34	0	1	1	1	0
2071	6.68	0	0	1	1	0
2074	6.63	1	1	1	1	0
2239	6.75	1	0	0	0	0
2257	6.51	0	0	1	1	0
2265	6.50	0	0	1	1	0

*^a^* The prioritized candidates are marked in light green.

**Table 3:** Average binding-site volumes (mean ± SD), calculated using CAVER Analyst over the final 20 ns of the MD trajectories, together with redocked CNN_VS values and differences relative to the initial docking scores. The apo-like reference volume, calculated over the same final 20 ns window, was 2584.32 ± 721.76 Å^3^.

Compound	Average Volume (Å^3^)	Δ_apo_ Volume (Å^3^)	Redocked CNN_VS	Δ Redocked CNN_VS
Aprepitant	1373.81 ± 194.79	−1210.51	7.51	0.62
Buclizine	2077.33 ± 526.92	−507.00	5.39	−1.05
Harringtonine	1307.03 ± 285.62	−1277.30	5.61	−1.02
Mavorixafor	1538.94 ± 227.36	−1045.38	6.52	−0.87
Mequitazine	1969.57 ± 220.45	−614.75	5.54	−1.19

## Data Availability

All data related to the analyses performed at different stages of this study, including the three-dimensional structures of the protein and ligand, as well as the input and output files associated with them, are freely available in the public GitHub repository (https://github.com/rocco-b/Delta-Opioid-Receptor-Agonists-Docking-and-MD-data, accessed on 31 March 2026).

## Supplementary Materials

The following supporting information can be downloaded at: https://www.mdpi.com/article/10.3390/ijms27093823/s1.
