# Supplementary material for: Structure-Guided Repurposing of Approved Drugs Identifies Aprepitant and Mavorixafor as Putative δ-Opioid Receptor Agonist Candidates
Source: Int J Mol Sci. 2026 Apr 25;27(9):3823. doi: 10.3390/ijms27093823 (PMC13163808; doi:10.3390/ijms27093823)
Supplement: Supplementary file 1 [file ijms-27-03823-s001.zip › ijms-4269505-supplementary.pdf]

International Journal of
Molecular Sciences

MDPI

Supporting information

Structure-Guided Repurposing of Approved Drugs Identifies Aprepitant and Mavorixafor as Putative δ-Opioid Receptor Agonist Candidates

Rocco Buccheri [†], Carlo Reale [†], Alessandro Coco, Carmela Parenti, Lorella Pasquinucci *, Antonio Rescifina *

Department of Drug and Health Sciences, University of Catania, Viale A. Doria 6, 95125 Catania, Italy; rocco.buccheri@unict.it (R.B.); carlo.reale@phd.unict.it (C.R.); alessandro.coco@phd.unict.it (A.C.); carmela.parenti@unict.it (C.P.)

* Correspondence: lorella.pasquinucci@unict.it (L.P.); antonio.rescifina@unict.it (A.R.)

† These authors contributed equally to this work.

Table of Contents

Figure S1. Two-dimensional structures of the 20 active molecules used in virtual screening active against decoys. S2

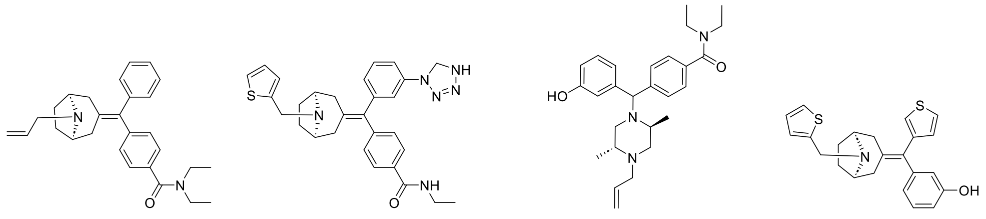

Compound **21** | Compound **23** | (+)-BW373U86 | Compound **24**

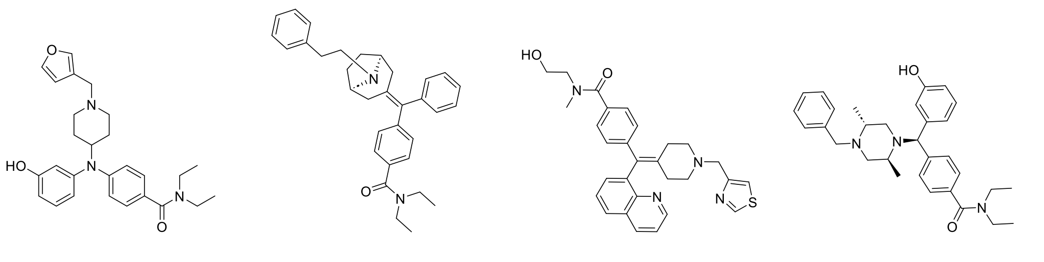

Compound **29** | Compound **22** | ADZ7268 | DPI-287

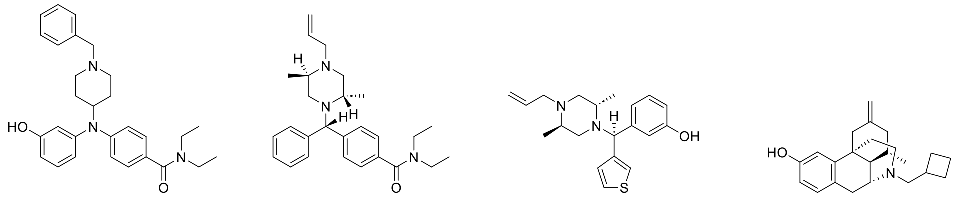

Compound **30** | SNC-162 | Compound **4** | Xorphanol

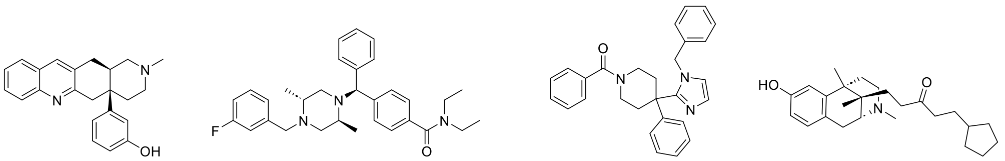

TAN-67 | DPI-221 | Compound **38** | WIN44,441

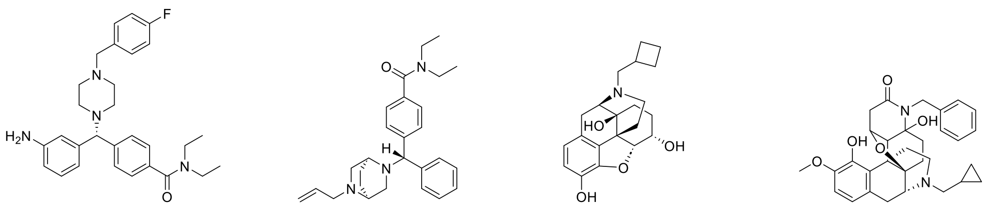

AZD2327 | Compound **9** | Nalbuphine | Oxazatricyclodecane (**2a**)

Figure S1. Two-dimensional structures of the 20 active molecules used in virtual screening against the decoy set.
